# Optimizing the Functional Properties of Starch-Based Biodegradable Films

**DOI:** 10.3390/foods12142812

**Published:** 2023-07-24

**Authors:** Theofilos Frangopoulos, Anna Marinopoulou, Athanasios Goulas, Eleni Likotrafiti, Jonathan Rhoades, Dimitrios Petridis, Eirini Kannidou, Alexios Stamelos, Maria Theodoridou, Athanasia Arampatzidou, Alexandra Tosounidou, Lazaros Tsekmes, Konstantinos Tsichlakis, Giorgos Gkikas, Eleftherios Tourasanidis, Vassilis Karageorgiou

**Affiliations:** 1 Department of Food Science and Technology, International Hellenic University, P.O. Box 141, 57400 Thessaloniki, Greece; thfrangopoulos@gmail.com (T.F.); amarinop@food.teithe.gr (A.M.); agoulas@ihu.gr (A.G.); likotraf@ihu.gr (E.L.); rhoadesjonathan@gmail.com (J.R.); dpmonas@gmail.com (D.P.); r3na95@gmail.com (E.K.); stamelosalexios@gmail.com (A.S.); theodoridoy1992@gmail.com (M.T.); sisi.aramp@gmail.com (A.A.); tosounidoualexandra21@gmail.com (A.T.); lazoc97@gmail.com (L.T.); dinostsichlakis@gmail.com (K.T.); 2 A. Hatzopoulos SA, Stadiou 21, Kalohori, 57009 Thessaloniki, Greece; gkikas@hatzopoulos.gr (G.G.); r_d@hatzopoulos.gr (E.T.)

**Keywords:** starch, montmorillonite, packaging films, definitive screening design, optimization, antimicrobial ability, biodegradability, industrial handling

## Abstract

A definitive screening design was used in order to evaluate the effects of starch, glycerol and montmorillonite (MMT) concentrations, as well as the drying temperature, drying tray type and starch species, on packaging film’s functional properties. Optimization showed that in order to obtain films with the minimum possible thickness, the maximum elongation at break, the maximum tensile strength, as well as reduced water vapor permeability and low opacity, a combination of factors should be used as follows: 5.5% wt starch concentration, 30% wt glycerol concentration on a dry starch basis, 10.5% wt MMT concentration on a dry starch basis, 45 °C drying temperature, chickpea as the starch species and plexiglass as the drying tray type. Based on these results, starch films were prepared, and fresh minced meat was stored in them for 3 days. It was shown that the incorporation of MMT at 10.5% wt on a dry starch basis in the packaging films led to a decreased mesophilic and psychrotrophic bacteria growth factor compared to commercial packaging. When assessed for their biodegradability, the starch films disintegrated after 10 days of thermophilic incubation under simulated composting conditions. Finally, to prove their handling capability during industrial production, the starch films were rewound in a paper cylinder using an industrial-scale rewinding machine.

## 1. Introduction

In recent years, a global turn is evident towards the use of non-petroleum-based packaging materials with biodegradable properties made from renewable sources for a variety of applications. The study of finding potential candidates to replace conventional non-biodegradable materials is not new and has proved to be a challenging endeavor because of the unique mechanical and practical advantages that come with the use of petrol-based polymers due to their variety of compositions, which enable the manufacture of end products with great mechanical properties and versatility [1,2,3].

A variety of strategies has been developed in the context of evolving alternatives to petrol-based polymers for packaging materials during the last decade. Among them, polysaccharides like cellulose derivatives and plant gums have been studied intensively [4]. Polylactic acid (PLA), as a biobased, recyclable material for packaging applications, has piqued the interest of the research community and industrial users due to its high efficiency and low cost [5]. Due to its availability, variety and versatility, however, starch is by far the most promising candidate for replacing petrol-based plastics with biodegradables [6]. Among the benefits of utilizing starch for packaging applications is its complete biodegradability [7] and its availability from various low-cost sources that are unfit for human consumption [8,9].

Starch, organized into unique starch granule structures, is the main polysaccharide in plants and is composed of linear amylose and highly branched amylopectin [10]. Although generally insoluble in water, when starch granules are heated in excess water at a temperature specific to each starch species, they absorb water and swell, leading to the disruption of the granule structure and the formation of a continuous polymeric matrix through the leached out amylose, which holds the gelatinized amylopectin embedded within it [11,12]. This transformation that starch granules undergo in the presence of water at elevated temperatures is called wet gelatinization.

In starch granule gelatinization, the transition from a crystalline to an amorphous physical state is basically irreversible. In a time- and condition-dependent process subsequent to the loss of granule crystallinity, water molecules that are bound in the amylose matrix are released, and amylose returns to its crystalline state through a reaction known as retrogradation [13]. Retrogradation of amylose has “negative” effects on the matrix formed after gelatinization. More precisely, in the case of starch film formulation, it causes brittleness and low elasticity due to intermolecular forces [1]. Counteracting and delaying the retrogradation of starch film formulations is achieved by the addition of plasticizers, most commonly glycerol [14], but also other alternative polyols [15].

Due to the physical nature of starch, especially following gelatinization, the materials that are formed either as films or as any other shape are susceptible to degradation, which is caused and enhanced by the materials’ high water vapor permeability [16] and poor mechanical properties [17]. Moreover, differences in biodegradability occur between starch species from different plant sources [18].

One very promising approach for improving the mechanical and physical properties of starch-based biodegradable films is the incorporation of nanoclay particles as nanofillers in the amylose matrix [19]. Montmorillonite (MMT), the most commonly used nanoclay, has several advantages that include its low cost, abundance, large surface area (760 m^2^/g) and high cation-exchange capacity [19,20]. These types of nanoclays consist of quasi-2D silicate sheets ordered in stacks with a single layer of an octahedral sheet of aluminum or magnesium oxide between two tetrahedral silica layers. The ease with which nanoclays form interlayer complexes with organic molecules, replacing metal ions (Na^+^, Li^+^, Mg^+^, Ca^2+^), is another beneficial property of these materials [8,20]. Starch–clay nanocomposites are obtained either by intercalation in solution or by melt intercalation, and once achieved, the starch-based material that is formed exhibits improved mechanical and physical properties [21]. Rates of nanoclay incorporation in the literature range up to 25% wt on a dry starch basis and, more specifically, from 1–7 [22], 0–10 [19] and 3–25% wt [8].

Nanocomposite biodegradable starch films are obtained in many ways, as discussed in the relevant literature. Among them, extrusion and casting are by far the most prominent methods [23,24,25], with the latter being an easier process to handle and operate, allowing for more flexibility to alter process conditions. As compared to extrusion, however, casting falters with regards to limitations on continuous production [25]. Casting is a complicated method that can lead to starch films with varying mechanical and physical properties, due to a diversity of factors regarding either the filmogenic thermoplastic solution ingredients or the process conditions that may be altered [2]. 

Starch, being the main film-forming material, is a dominant factor affecting film quality, since as it increases, amylose also increases, leading to enhanced intermolecular forces between the components of the thermoplastic starch film [26,27]. Starch, and particularly thermoplastic starch, is already used in commercial biodegradable packaging [28].

Plasticizers also affect the films’ mechanical and physical properties to a great extent. The occurrence of undisrupted intramolecular hydrogen bonding between amylose molecules causes the formation of rigid, brittle films with high tensile strength and low elongation at break values [29]. Due to their interaction with water, the presence and quantity of plasticizers within the amylose matrix affect the film’s mechanical properties in a concentration-dependent manner by increasing intermolecular spacing and reducing intramolecular hydrogen-bonding forces [30]. This bound water within the film matrix, induced by the presence of plasticizers, concomitantly affects the film’s exhibited rigidity and glass transition temperatures [31]. With regards to the food industry, the most commonly used secondary plasticizer is glycerol, due to its low toxicity and safe nature. Although plasticizers are necessary for obtaining films with enhanced elasticity, it is crucial to regulate their presence with respect to the rest of the film components, because increased concentrations lead to weak films with low tensile strength and durability. These are hydrophilic, with poor water vapor barrier properties [32]. In the relevant literature, a variety of glycerol addition rates have been studied, ranging between 0 and 40% wt on total dry starch content [30]. 

As referred to previously, film formation mainly depends on the amylose content of starch, which in turn varies depending on the starch species. Amylose content is greater in legume starches (30–35% wt) [33], followed by maize and potato starches (22–29 and 21–26% wt, respectively) [18]. Post casting, the choice of film drying temperature, air humidity and drying time also play an important role in the physicochemical state of the amylose matrix, influencing its degree of crystallinity [34].

The aim and novelty of the present study lays in its effectiveness evaluation, through a parametric analysis method, of different quantitative and qualitative factors regarding the mechanical, physicochemical and barrier properties of starch-based biodegradable films. Simultaneously, it offers a technique for determining the optimum concentrations and levels of factors for the final product in a desired property-dependent manner. Furthermore, for the first time in the relevant literature, this type of film was used for storing perishable food (fresh minced meat) and was tested in terms of handling at an industrial scale.

## 2. Materials and Methods

### 2.1. Μaterials

Legumes and rice were purchased from Agrino (Thessaloniki, Greece). Glycerol (99% Purity) was purchased from Carlo Erba reagents (Cornaredo, Italy). Organically modified montmorillonite nanoclay (modified with dimethyl, benzyl, hydrogenated tallow, quaternary ammonium (2MBHT) with a cation-exchange capacity of 125 meq/100 g, 99% purity) was purchased from Nanocel LG (Punjab, India). Plate count agar (PCA) was purchased from NeoGen (Lansing, MI, USA). Fresh minced beef (M. biceps femoris, M. semitendinosus, M. semimembranosus) was purchased from a local market at 48 h post-mortem.

### 2.2. Methods

#### 2.2.1. Experimental Design

In order to examine the different factor levels of the mechanical, barrier and optical properties of biodegradable starch films studied herein, definitive screening design (DSD) methods were applied using the Design of Experiments mode in JMP 17 software by SAS™. The required test runs were estimated to be 18, resulting in the existence of two categorical and four numerical factors (2k + 2, k = 6), with the addition of four ghost runs with two center points fixed on the mean values of continuous factors (Table 1). Analysis of variance (ANOVA) was applied to evaluate the significance of all coefficients of the models, and the model adequacy was determined by the lack-of-fit test, coefficient of determination (R^2^), and adjusted R^2^ value at a 95% confidence level. It was considered that the tensile strength, elongation at break (%), barrier properties and thickness are essential qualities to produce films with suitable physical integrity for handling, packaging and transport. Experimental values of the independent variables presented in Table 2 were selected based on preliminary experiments.

#### 2.2.2. Starch Extraction from Chickpeas and Rice

Starch was isolated from chickpeas at a pilot scale according to the following procedure: Dry chickpeas were soaked in an excess of sodium metabisulfite 1.5% wt solution in soft water (≈10 μS/cm) for 48 h at ambient temperature (18–22 °C). Subsequently, the impregnated seeds were separated from the metabisulfite solution and wet ground to a pulp using an APEX 314S-SS hammer mill (APEX Construction LTD, Somerset, UK) fitted with a 2 mm screen. During grinding, soft water in a 4:1 weight ratio to the soaked chickpeas was added. Chickpea peals were concomitantly separated from the pureed legumes through a refining 0.5 mm screen skin and a seed separator/finisher (Henri Biaugeaud, Mérignac, France). Congo red dye was used in order to identify the damaged starch granules. The results showed that the damage to starch granules was insignificant. Following skin removal, the chickpea pulp was wet sieved through a custom-made vibrating sieve separator, where smaller fiber, flesh and skin pieces were removed through subsequent 0.5 mm and 53 μm sieves. Finally, starch was isolated and purified through four (4) successive sedimentation steps in water, where impurities in the surface layer of the sediment, were pneumatically removed following each sedimentation. The final chickpea starch preparation, resulting from the previously described process, was later dried in a laminar air flow tray dryer/oven (APEX Construction LTD, UK). The moisture and protein contents of the chickpea starch were 10.68 ± 0.35% and 1.26 ± 1.08%, respectively, determined by the gravimetric and Kjeldahl methods. The yield achieved was in the range of 20–25%.

The extraction of starch from rice was performed following the method of Cardoso et al. (2007) with some modifications [35]. In detail, in order to loosen the rice grains and prepare them for grinding, polished dry broken rice was soaked in an excess of sodium metabisulfite 1.5% wt solution in soft water (≈10 μS/cm) for 48 h at ambient temperature (18–22 °C). Subsequently, the impregnated seeds were separated from the metabisulfite solution and wet ground to a pulp using an APEX 314S-SS hammer mill (APEX Construction LTD, UK) fitted with a 2 mm screen. During wet grinding, in order to assist the process, a 0.2% wt NaOH solution in soft water in a 4:1 weight ratio to the soaked rice was added, and the grinding temperature was strictly monitored and maintained at 20 °C to minimize alkaline gelatinization of starch. Rice pieces that were not fully ground were concomitantly separated from the rice puree through a refining 0.5 mm screen skin and a seed separator/finisher (Henri Biaugeaud, France). Congo red dye was used to identify the damaged starch granules, and the results showed that the damage was insignificant. Consequently, the rice pulp was wet sieved through a custom-made vibrating sieve separator, where smaller fiber, flesh and skin pieces were removed through subsequent 0.5 mm and 53 μm sieves. The mixture, post-sieving, was agitated continuously for 4 h at 20 °C before being allowed to settle at 8 °C for 12 h. The resulting liquor and the dark impurities in the surface layer of the sediment were pneumatically removed, and the sediment was resuspended and agitated for 4 h at 20 °C in a 0.2% wt NaOH solution in a 1:10 weight ratio before being allowed to settle again at 8 °C. Removal of the liquor and impurities from the surface layer of the sediment, along with the NaOH treatment and sedimentation steps, were repeated 2 more times. Finally, the resulting starch was washed with soft water to nearly neutral pH, collected and cleaned each time with sedimentation and pneumatic removal of the surface layers, before drying in a laminar air flow tray dryer/oven (APEX Construction LTD, UK). The moisture and protein contents were 11.06 ± 0.46% and 6.88 ± 6.87% wt, respectively, determined by the gravimetric and Kjeldahl methods. The starch yield achieved was in the range between 25 and 28%.

#### 2.2.3. Preparation of Thermoplastic Starch–Nanoclay Films

The thermoplastic starch films were prepared by a simple solution-mixing procedure based on methods described in the literature with some modifications [36,37,38]. Briefly, different concentrations of between 4 and 7% on a dry weight basis of the two starch types were dispersed in distilled cold water containing different concentrations of glycerol ranging from 20 to 50% wt on a dry starch basis. MMT dispersions in distilled water in concentrations of up to 1% wt were stirred overnight and sonicated for 15 min at 15,000 kHz before being added to the starch–glycerol mixture in varying quantities in order to obtain different final concentrations of MMT (1–20% wt on a dry starch basis) in the thermoplastic starch solutions. The resulting dispersion was stirred initially for 30 min at 80 °C to achieve complete starch gelatinization, and subsequently, the temperature was raised to 90 °C for 15 min for inducing nanoclay intercalation. Films were obtained by hot casting on poly-methyl 2-methylpropenoate (plexiglass) and poly-tetrafluoroethylene (teflon) plates with dimensions 10 cm × 6.8 cm or 18 cm × 11 cm and dried in a laminar air flow oven at a range of different temperatures between 30 and 60 °C for ~24 h. Subsequently, the films were peeled from the plates and stored for 10 days at room temperature for moisture equilibration before further analyses. 

#### 2.2.4. Thickness

The films’ thickness was measured with a digital electronic caliper (TESA, North Kingstown, RI, USA), and a total of 12 measurements were recorded for each film. 

#### 2.2.5. Mechanical Properties

The films’ mechanical properties, specifically the elongation at break and tensile strength, were determined using the stress–strain graphs resulting from tension tests. The tensile properties of the film samples were determined according to the ASTM D882-10 standard test method [39]. Briefly, film specimens 10 cm in length and 1.5 cm width were cut with a medical scalpel, and their mechanical characteristics were evaluated with a TA-XT Plus Texture Analyzer (Stable Microsystems Ltd., Godalming, UK) with a cross-head speed (rate of grip separation) of 50 mm/min. For each film formulation, three replicate films were prepared, and from each film, five replicate samples were tested. In total, 15 replicate measurements were performed at ambient temperature (23 °C) for each different formulation.

#### 2.2.6. Water Vapor Permeability (WVP)

The films’ WVP was determined gravimetrically based on the method proposed by Mali et al. (2002) and the ASTM E96/E96M-05 desiccant method with some modifications [4,40]. Films were used to seal the circular opening area of permeation cells, which were subsequently placed at 35 °C in a desiccator. The relative humidity (RH%) inside the desiccator was fixed at 75% (R_1_) with a saturated sodium chloride solution, whereas inside the permeation cells, it was maintained at 0% (R_2_) with dry silica gel, generating a constant RH difference between the two sides of the film. Water vapor transport was determined from the weight gain of the silica gel. After equilibrium, eight weight gain measurements were recorded over a 48 h period to the nearest 0.001 g and plotted as a function of time. The slope of each line was calculated by linear regression (r^2^ > 0.99), and the water vapor transmission rate (WVTR) was calculated from the straight-line slope divided by the cell opening area sealed by the starch films. WVP was calculated as:

$$WVP=\frac{WVTR}{S\left(R_{1}-R_{2}\right)d}$$

where S is the saturation vapor pressure of water (Pa) at the test temperature (35 °C), and d is the film thickness. Under the test conditions, the vapor difference driving force [S (R_1_ − R_2_)] was 3169.65 Pa.

#### 2.2.7. Optical Parameters

The films’ optical parameters were determined using a non-contact imaging spectrophotometer (MetaVue VS3200, X-Rite, Grand Rapids, MI, USA) and Color IQC 10 software (X-Rite, Grand Rapids, MI, USA). The opacity index was estimated by measuring the contrast of a film specimen over a white calibration plate and then over a black calibration plate. A D65 (daylight) CIELab scale was used to determine the color parameters of L* (from black (0) to white (100)), a* (from green (−128) to red (127)) and b* (from blue (−128) to yellow (127)). The white plate color standard (L* = 94.8, a* = −0.78, b* = 1.43) was used as the background for determining the film color.

#### 2.2.8. Optimization

An optimization and desirability function approach was utilized to optimize the multiple responses. It was considered that tensile strength, elongation at break, WVP, thickness and opacity properties are essential to produce packaging films with adequate physical attributes. The film composition that met the conditions of the maximum tensile strength, the maximum elongation at break, the lowest possible thickness, the minimum WVP and the minimum opacity was determined using JMP 17 software by SAS™.

#### 2.2.9. Antimicrobial Activity

The antimicrobial activity of the biodegradable starch films for packaging applications was tested on fresh minced meat against natural microbiota (mesophilic and psychrotrophic bacteria). Biodegradable starch films produced according to the formulation suggested by the optimization method were sterilized with UV light (15 min, each surface). Minced meat samples (30 g) were placed in packages made of two films sealed with starch glue, and the packages were stored in the refrigerator (4 °C) for 3 days before further analyses. Three treatments were tested, namely, a thermoplastic starch film package (TS), a thermoplastic starch film package with MMT (TS + MMT) and a commercial food package made of LDPE (commercial). Two packages (subjects) were assigned to each treatment, and the analyses were performed over 3 consecutive days plus the 0 (initial) day. Serial dilutions and the pour plate method were used with plate count agar (PCA), and after incubation at 30 °C for 72 h for the mesophilic bacteria and at 4 °C for 10 days for the psychrotrophic bacteria, the CFU/g was recorded. Tests were performed in duplicate. Results were expressed as the growth factor, i.e., the difference between the log cfu/g of each day and the log cfu/g of day 0. The experiment was conducted twice.

A repeated-measures analysis of variance (rANOVA) including a between-subjects effect (two experimental pouches) and a fixed between-subjects factor (treatments) was applied. This design was selected to evaluate whether and how the different treatment affected every response, whether there was a different performance in the responses over the preservation time and whether a significant interaction developed between treatment and preservation time, i.e., whether at least one treatment affected the responses differently over a period of time as compared to the others. The results are depicted graphically as mean changes with 95% confidence intervals based on the error mean square of rANOVA. Pairwise indicates that values whose intervals do not overlap denote significant difference.

#### 2.2.10. Biodegradability

The films’ biodegradability was assessed according to the ISO 20200:2015 [41]. Briefly, starch films produced according to the formulation suggested by the optimization method were cut into 25 mm × 25 mm specimens and buried in synthetic solid waste composed of 45% wt dry mass (sawdust 40% wt, rabbit feed 30% wt, ripe compost 10% wt, corn starch 10% wt, saccharose 5% wt, corn seed oil 4% wt and urea 1% wt) and 55% wt de-ionized water that was placed into plastic boxes. To simulate thermophilic incubation, the boxes were placed in an incubator operating at 58 °C and 60% RH. Every day, the boxes were weighed, and water was added to restore the initial mass, followed by mixing of the composting matter. Every 2 days, the films were removed, or their pieces were collected to the best extent possible, cleaned from the composting matter without breaching the integrity of the films and photographed.

#### 2.2.11. Industrial-Scale Handling

A sheet of biodegradable starch film (110 cm × 38 cm) that was produced according to the recipe suggested by the optimization method was cut into specific dimensions (90 cm × 30 cm) in order to fit the technical specifications of the rewinding machine (CB 600 S, Euromac, Formigine, Italy). During the rewinding process of the commercial film, the machine was stopped, and the commercial film was cut in order to attach the biodegradable starch film in the middle (between unwinding and rewinding) section. The two films were joined together using a standard double-sided adhesive tape. The rewinding process was continued, and the biodegradable starch film was rewound in a paper core with a 77 mm internal diameter.

## 3. Results and Discussion

### 3.1. Thickness

**Figure 1:**
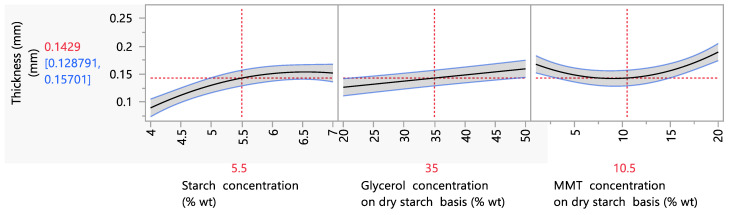
Mean main effects of starch, glycerol and MMT concentration on film thickness.

In general, the thickness of the films in the present study varied between 0.09 and 0.25 mm. Films of similar thickness were also produced in several studies. More specifically, in the study of Giannakas et al. (2022), biodegradable films of thicknesses between 0.1 and 0.3 were investigated [5]. Also, in the work of Henrique et al. (2007), starch biodegradable films of 0.08 to 0.12 mm thickness were investigated [42]. Parametric analysis data showed that the starch, glycerol and ΜΜΤ concentration had a statistically significant (*p* < 0.05) effect on the film thickness, and more specifically, an increase in the starch or glycerol content led to an increase in the film thickness from 0.09 mm to 0.16 mm when the starch concentration increased from 4 to 7% wt (Figure 1). The same result was observed also in the literature and was attributed to the higher water absorption due to the hygroscopic character of both gelatinized starch and glycerol, which acts as a plasticizer, as well as to the general increase in the content of solids that accompanies the increase in the starch concentration in the film solution and, consequently, in the film [43,44,45,46]. Specifically, Bidari et al. (2023) reported that increasing the starch concentration in a biodegradable film formulation from 2.3 to 3.7% wt led to an increase in the film’s thickness of almost 50% [46]. Furthermore, the addition of MMT caused an increase in the film’s thickness, and, more specifically, in the range of 1–20% wt MMT, the thickness increased from 0.17 to 0.26 mm. This was explained by the large particle size of the nanoclay [47] but also by the increase in the solid content of the film [48]. These results were also observed in the aforementioned studies when nanosilicates were incorporated into starch films. Specifically, Muller et al. (2012) [49] reported that the incorporation of MMT into a starch biodegradable film formulation in concentrations between 0 and 5% wt on a dry starch basis increased the film thickness from 0.39 to 0.52 mm.

### 3.2. Mechanical Properties

#### 3.2.1. Elongation at Break

**Figure 2:**
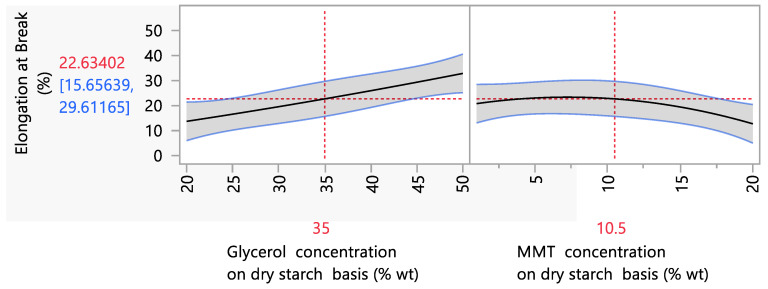
Mean main effects of glycerol and MMT concentration on the elongation at break.

Parametric analysis data showed that the glycerol and MMT concentration had a statistically significant (*p* < 0.05) effect on the elongation at break values (Figure 2). More specifically, increasing the glycerol concentration led to an increase in the elongation at break. This trend is in accordance with the literature, and more specifically with the studies of Mansour et al. (2020) and Marques et al. (2019) in which, independently of nanoclay concentration, starch biodegradable films with increased glycerol concentrations presented higher values in the elongation at break [2,9]. Physicochemically, this is explained by the plasticizing effect of polyols (more specifically, glycerol) in the amylose matrix of thermoplastic starch. The fact that glycerol consists of three carbon atoms, which enables the glycerol molecule to enter into the polymer matrix more easily, leading to a higher mobility, a higher water absorption and a more efficient plasticizing effect, explains the higher reduction in intramolecular forces, the increase in the space between starch chains and, consequently, the more elastic structure with higher extensibility [50,51].

However, a difference between the present work and the literature is the impact of MMT incorporation on the film matrix at the elongation at break. Ιt is remarkable that the addition of MMT at concentrations between 1 and 20% wt on a dry starch basis did not show a significant effect (*p* < 0.05) on the elongation at break values. This is in contrast to the existing literature, which has shown that an increase in the nanoclay concentration resulted in lower elongation at break values. More specifically, the incorporation of MMT presented a remarkably negative impact on the elongation at the break of starch films, and more specifically, the elongation at the break of starch films with 5% wt MMT on a dry starch basis was 3.2% compared with starch films without MMT, for which the elongation at break was 10.7% [52]. At the same time, films prepared with 4% wt starch concentration, 30% wt glycerol and 15% wt MMT on a dry starch basis presented an elongation at the break of 2.4% compared to starch films without the incorporation of MMT, for which the elongation at the break was 40% [53]. This effect has been explained by the strong interaction between nanocomposites and the polymer matrix through intercalation or exfoliation, which enhances the mechanical resistance but decreases flexibility [52,53,54,55]. In the present work, MMT concentrations between 1 and 20% wt on a dry starch basis significantly reduced the elongation at the break of starch films only after 10% wt MMT on a dry starch basis was added. Furthermore, this loss in extensibility (from 20% to 12%, when the MMT concentration increased from 1 to 20% wt on a dry starch basis) is significantly lower than that in the aforementioned literature. The fact that the negative effect of the addition of MMT on the elongation at break was absent in our study may be due to the enhanced nanoclay intercalation into the amylose matrix. The nanoclay that was used was modified with a quaternary ammonium salt, specifically bis(hydrogenated tallow alkyl)dimethyl salt. According to the literature, alkylation or quaternization of tertiary amines with alkyl halides has a positive effect on nanoclay’s thermomechanical properties [56], and thus more efficient exfoliation/intercalation.

#### 3.2.2. Tensile Strength

**Figure 3:**
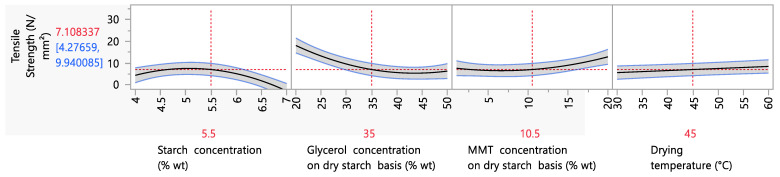
Mean main effects of starch, glycerol and MMT concentration and drying temperature and on the tensile strength.

Parametric analysis data for the tensile strength showed that the starch, glycerol and MMT concentrations and the drying temperature had significant effects (*p* < 0.05) (Figure 3). Regarding the tensile strength, it is remarkable that increasing the starch content led first to an increase, until the starch content reached 5.5% wt (maximum value of 7.23 N/mm^2^), and then to a decrease in the tensile strength. The first trend is in accordance with the available literature, in which an increase in starch content is correlated with increasing tensile strength values—a fact that is explained by the strengthening of the amylose matrix [2]. As for the second trend (decreasing tensile strength values at 5.5 to 7% wt starch concentration), this could be attributed to the enhanced water retention capacity of the amylose matrix, due to the increased starch concentration [57], which led to a higher film moisture content, and hence a lower resistance to stress.

Regarding the effect of the glycerol concentration on the tensile strength, a decreasing trend was observed as the glycerol concentration increased, as expected. Glycerol, which acts as a secondary plasticizer, interacts with water, causing a reduction in intramolecular forces and, consequently, a reduction in the tensile strength values and an increase in the elongation at break values [9,50]. Regarding the MMT concentration, it is evident that increasing the MMT concentration caused an increase in the tensile strength values, reaching a maximum of 11.35 N/mm^2^ when the MMT concentration was 20% wt. This is in accordance with the literature and the incorporation of nanoclays from different sources or different types that act as fillers in junction zones, strengthening the amylose matrix [8,9].

As mentioned above, MMT enhances the mechanical properties of polymeric films through the process of intercalation within the amylose network, creating intra- and inter-molecular interactions, and thus enhancing the strength and structure of the resulting films. Still in question is the range of nanoclay concentrations over which this enhancement of mechanical properties is observed, as well as whether this enhancement applies to all mechanical properties. Based on Figure 3, the increase in tensile strength values is initiated after 10.5% wt (at this level, the tensile strength corresponds to 7.20 N/mm^2^) and reaches a maximum at 20% wt (at this level, the tensile strength value equals ~12 N/mm^2^). This trend is in accordance with the available literature, particularly the study of Romero-Bastida et al. (2015) and Kochkina et al. (2021), who reported an increase in the tensile strength in starch films, from 6.2 (0% wt MMT on a dry starch basis) to 15.2 N/mm^2^ (5% wt MMT on a dry starch basis) and 4 (0% wt MMT on a dry starch basis) to 13 N/mm^2^ (15% wt MMT on a dry starch basis), respectively [52,53].

### 3.3. Water Vapor Permeability (WVP)

**Figure 4:**
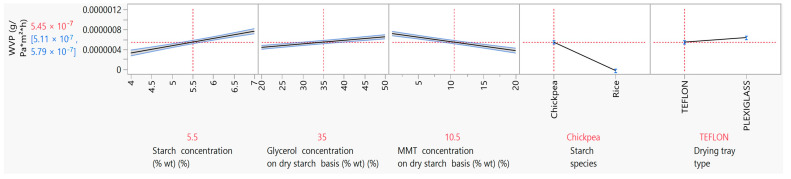
Mean main effects of starch, glycerol and MMT concentration, starch species and drying tray type on WVP.

Barrier properties, particularly the film’s resistance to moisture, is a very important property in materials intended for use in food packaging. Biodegradable films based on natural materials have poor barrier properties in general, because natural materials are hydrophilic [9]. The WVP is an indicator of the film’s water barrier properties and measures the rate of moisture transmission from a high-moisture environment to a low-moisture environment through the film [4]. The vapor transmission is guided by both vapor diffusion through the film and the vapor molecules’ attraction from the hydrophilic parts of the film matrix [9]. In the present study, parametric analysis results showed that the WVP was influenced significantly (*p* < 0.05) by the starch, glycerol and MMT concentration, the starch species and the drying tray type (Figure 4). More specifically, it was observed that an increase in both the starch and glycerol concentration lead to an increase in the WVP. Taking into account that films with higher starch and glycerol content also had a higher moisture content it makes sense that the hydroxyl groups of glycerol, on the one hand, and the increase in the starch concentration, on the other, increased hydrophilicity, attracted water molecules and, consequently, increased permeability to vapor [4,44]. The negative effect of glycerol and starch concentration on the WVP has also been observed by Mali et al. (2002) [4]. In their study, a 0.7% increase in the starch concentration led to a ~22% increase in the WVP, and a 1.3% increase in the glycerol concentration led to a ~32% increase in the WVP. The negative impact of the glycerol concentration on the WVP of starch films prepared with 3% wt cassava starch was also observed in the work of Müller et al. (2008) [44]. More specifically, increasing the glycerol concentration from 25 to 35% wt led to an increase of the WVP from 2.46 × 10^−7^ to 4.42 × 10^−7^ g·Pa^−1^·h^−1^·m^−1^. In the present study, the same trend in the WVP of films with 3% wt chickpea starch was observed, but with lower values (1.08 × 10^−7^ and 1.79 × 10^−7^ g·Pa^−1^·h^−1^·m^−1^ for films with 25 and 35% wt glycerol on a dry starch basis, respectively).

The incorporation of MMT into the thermoplastic starch solution for film casting applications was not only intended to solve problems related to the poor tensile properties of starch films, but also to improve the barrier properties, particularly the barrier against water vapor [19]. In the present study, it was evident that increasing the MMT concentration in starch films led to lower (*p* < 0.05) values for the WVP (Figure 4). More specifically, films prepared with 5.5% wt chickpea starch and 1% and 20% wt MMT on a dry starch basis had WVP values of 7.17 × 10^−7^ and 3.73 × 10^−7^ (g·Pa^−1^·h^−1^·m^−1^), respectively. Similar results were also observed in the study of Tang et al. (2008), in which MMT incorporation into corn starch films at 9% wt on a dry starch basis led to an one-fold decrease in the WVP compared to the control [58], and in that of Mansour et al. (2020b), in which MMT incorporation into 6.5% wt pregelatinized maize starch films from 0 to 20% wt on a dry starch basis led to a decrease in the WVP from 18.9 to 12.9 ((g/day)/m^2^) [8].

### 3.4. Optical Parameters

#### 3.4.1. Opacity Index

**Figure 5:**
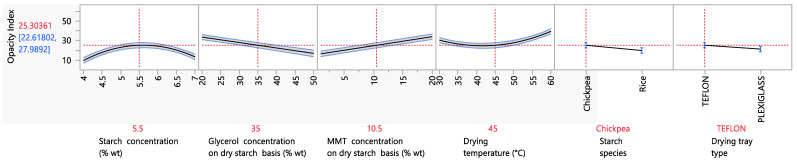
Mean main effects of starch, glycerol and MMT concentration, drying temperature, starch species and drying tray type on the opacity index.

The opacity index is an indicator of how transparent a product is. Generally, light transmission properties are very important in the food packaging industry, since they determine how well a consumer can see a product before its purchase, and they thus ultimately influence the acceptance of the product by the consumers. It is favorable for a packaging material to be transparent, and therefore, a biodegradable food packaging material that aims to replace the conventional synthetic plastic packaging should also present good optical properties. As can be seen from Figure 5, the opacity of starch biodegradable films with MMT was influenced by a number of experimental factors. Regarding the starch concentration, it can be seen that the opacity increased when the starch concentration ranged from 4 to 5.5% wt and then decreased between 5.5 and 7% wt. The initial increasing trend in the opacity index (or decreasing trend in the transparency) as the starch concentration in the thermoplastic solution increased from 4 to 5.5% (opacity index increased from ~10 to 30) can be explained by the increase in undissolved starch aggregates in the film. More specifically, because starch granule swelling, disruption, dissolution and gelatinization are not complete, starch ghost particles occur, which form aggregates after drying the films, eventually leading to increased light scattering, and thus opacity [59,60]. However, the following decreasing trend in the opacity index from ~30 to 10 when the starch concentration increased from 5.5 to 7% wt could presumably be attributed to the fact that the remaining ordered structure of the starch granules became so compact that it did not allow the particles to swell due to the increase in compressibility [61], and therefore the light subsequently scattered.

Regarding the influence of the glycerol concentration on the opacity index, it can be seen that increasing the glycerol concentration from 20 to 50% wt on a dry starch basis led to a nearly three-fold decrease in the opacity index (or increase in the transparency). This observation was also made in the study of Fu et al. (2021), in which increasing the plasticizer concentration caused a decrease in the opacity index of starch films as well. This was mainly attributed to the better starch gelatinization in the presence of a higher plasticizer concentration, resulting in lower quantities of undissolved starch ghost particles that scatter the light and increase opacity [60].

In the case of the MMT concentration, it can be seen that increasing the MMT concentration from 1 to 10.5% wt on a dry starch basis caused a ~two-fold increase in the opacity index, resulting in less transparent films. This observation is in agreement with the work of Slavutsky et al. (2012) [62], in which an increase in the MMT concentration from 1 to 10% wt on a dry starch basis resulted in an increase in the film opacity by double. For a dense material such as MMT to decrease the transparency of packaging films, however, it is expected that the method of incorporation plays an important role as well. More specifically, the MMT incorporation method used in the present work was intercalation, but exfoliation is a very common method of MMT incorporation as well, resulting in more transparent and clear films in some cases [62,63].

The film opacity increased as the drying temperature increased. When the film is dried at lower temperatures, the mobility of MMT particles decreases, inhibiting the aggregation of MMT particles and, therefore, resulting in less opaque films. Regarding the starch type, films prepared from chickpea starch were opaquer than films prepared from rice starch. Taking into consideration that the size of rice starch granules is 2–7 μm [64], whereas the size of chickpea starch granules is 10–30 μm [33], it is expected that chickpea granules will scatter more light and, therefore, result in opaquer films. Finally, the different material of the drying tray had an effect on opacity, with films cast in Teflon trays being opaquer that those cast in Plexiglass trays. A possible explanation for this is the more hydrophilic nature of Plexiglass; indeed, the side of the film that was in touch with the drying tray was smoother when the drying tray material was Plexiglass, and smoother surfaces are more transparent [65].

#### 3.4.2. Color Parameters

**Figure 6:**
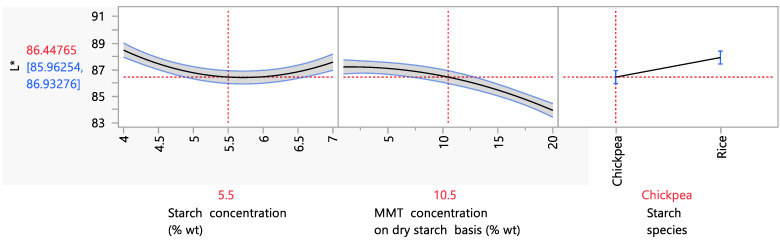
Mean main effects of the starch and MMT concentration and starch species on the L* value.

Since starch biodegradable films aim to replace conventional plastic packaging for food products, it is understandable that the film’s color properties are of great importance. The L* value is an indicator of the lightness and clarity of samples. As can be seen from Figure 6, the film’s L* value was affected by the starch and MMT concentration and by the starch species. More specifically, it was observed that as the starch concentration increased from 4 to 5.5% wt, a decreasing trend in the L* value was observed. Meanwhile, when the starch concentration increased from 5.5 to 7% wt, the L* value increased. These observations should be related to the results of Figure 5, more specifically to the effect of the starch concentration on the opacity. As has been shown in the literature, a connection exists between transparency and lightness values originating from the behavior of light transmittance on surfaces. As the density of a solution increases, the diffuse reflectance also increases [66], the transparency decreases, and the L* value thus decreases. The same observation was made by Ghasemlou et al. (2013) [67], for whom the transparency and L* value followed the same trend as the filmogenic solution density increased. In the study of Ramos da Silva et al. (2022), when the rice starch content of films increased from 3 to 5% wt, a significant decrease in the L* value from 94.1 to 93 was observed, which is in accordance with the present work, in which a similar decrease was observed (from 92.4 to 89) [68].

Regarding the MMT concentration’s effect on the L* value, a decreasing trend was observed, which became steeper after ~10% wt MMT concentration on a dry starch basis. More specifically, the L* decreased from 87.2 to 86.5 for MMT concentrations between 1 and 10.5% wt on a dry starch basis, and it decreased from 86.5 to 83.9 for MMT concentrations between 10.5 and 20% wt on a dry starch basis. The same trend was observed in the study by Hong et al. (2022), in which increasing the MMT content in the starch films from 1 to 5% wt on a dry starch basis caused a decrease in the L* value from 95.6 to 88.1. This fact was attributed to the increase in the film’s density due to the large particle size of MMT [69].

**Figure 7:**
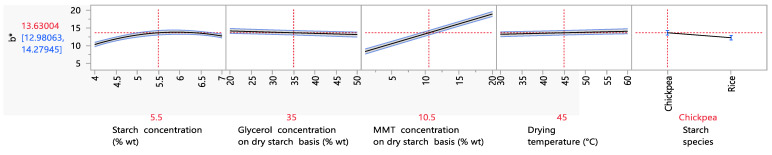
Mean main effects of the starch, glycerol and MMT concentration, drying temperature and starch species on the b* value.

The factors influencing the film’s b* value or yellowness are presented in Figure 7. An increase in the b* value from 1.7 to 6.5 was observed when the starch content was increased from 3 to 5% wt. This observation was also made in a study by Ramos da Silva et al. (2022). For the same starch content range, the b* value increased from 4.2 to 4.4. A possible explanation for this observation could be the migration of natural pigments (vitamin B group) onto the starch granules [68].

Furthermore, increasing the MMT concentration resulted in an increase in the film’s yellowness. More specifically, an increase in the MMT concentration from 1 to 5% wt on a dry starch basis led to an increase in the b* value from 8.1 to 10.1. This can be attributed to the yellowish color of the MMT powder itself. This increase, however, was much lower than the one reported in the work of Hong et al. (2022) for the same levels of MMT content. In this study, the b* value increased from 10.7 to 29.6 [69]. However, a direct comparison is not completely appropriate, due to differences in film preparation between the two studies.

### 3.5. Optimization

The optimization of the film thickness based on the statistically significant examined functional parameters (starch, glycerol and MMT concentration) was derived using multiple linear regression analysis based on the results. The linear regression equation (R^2^ = 0.78) is:

(1) $$\begin{array}{cc} Y=0.142+0.031 & \times \left(\frac{C_{Starch\;}-5.5}{1.5}\right)+0.017\times \left(\frac{C_{Glycerol\;}-35}{15}\right)\\ & +0.01\times \left(\frac{C_{MMT\;}-10.5}{9.5}\right)\\ & +\left(\frac{C_{Starch\;}-5.5}{1.5}\right)\times \left(\left(\frac{C_{Glycerol\;}-35}{15}\right)\times 0.015\right)\\ & +\left(\frac{C_{Starch\;}-5.5}{1.5}\right)\times \left(\;\left(\frac{C_{Starch\;}-5.5}{1.5}\right)\times -0.022\right)\\ & +\left(\frac{C_{MMT\;}-10.5}{9.5}\right)\times \left(\left(\frac{C_{MMT\;}-10.5}{9.5}\right)\times 0.036\right) \end{array}$$

The optimization of the mechanical properties and, more specifically, the elongation at the break based on the examined functional parameters (glycerol and MMT concentration) was derived using multiple linear regression analysis based on the results. The linear regression equation (R^2^ = 0.53) obtained from the experimental data is:

(2) $$\begin{array}{cc} Y=22.63+9.51 & \times \left(\frac{C_{Starch\;}-35}{15}\right)-4\times \left(\frac{C_{MMT\;}-10.5}{9.5}\right)\\ & +\left(\frac{C_{\mathrm{Glycerol}\;}-35}{15}\right)\times \left(\left(\frac{C_{MMT\;}-10.5}{9.5}\right)-5.12\right)\\ & +\left(\frac{C_{\mathrm{Glycerol}}\;-35}{15}\right)\times \left(\;\left(\frac{C_{\mathrm{Glycerol}\;}-35}{15}\right)\times 0.59\right)\\ & +\left(\frac{C_{MMT\;}-10.5}{9.5}\right)\times \left(\left(\frac{C_{MMT\;}-10.5}{9.5}\right)\times -5.89\right) \end{array}$$

The corresponding linear regression equation (R^2^ = 0.84) for the optimization of the tensile strength based on the examined functional parameters (starch, glycerol and MMT concentration and drying temperature) is:

(3) $$\begin{array}{cc} Y=7.11-3.59 & \times \left(\frac{C_{Starch}-5.5}{1.5}\right)-5.87\times \left(\frac{C_{Glycerol}-35}{15}\right)\\ & +1.41\times \left(\frac{Drying\;temperature-45}{15}\right)\\ & +2.62\times \left(\frac{C_{MMT}-10.5}{9.5}\right)\\ & +\left(\frac{C_{Starch}-5.5}{1.5}\right)\times \left(\left(\frac{C_{Glycerol}-35}{15}\right)\times 4.66\right)\\ & +\left(\frac{C_{Starch}-5.5}{1.5}\right)\times \left(\left(\frac{C_{Starch}-5.5}{1.5}\right)\times -6.24\right)\\ & +\left(\frac{C_{Glycerol}-35}{15}\right)\times (\left(\frac{C_{Glycerol}-35}{15}\right)\times 5.15)\\ & +\left(\frac{C_{MMT}-10.5}{9.5}\right)\times (\left(\frac{C_{MMT}-10.5}{9.5}\right)\times 3.21) \end{array}$$

The optimization of the WVP was based on the statistically significant examined functional parameters (starch, glycerol and MMT concentration, starch species and drying tray type). The linear regression equation (R^2^ = 0.97) is:

(4) $$\begin{array}{cc} Y=3.04\times 10^{-7} & +1.36\times 10^{-7}\times \left(\frac{C_{Starch\;}-5.5}{1.5}\right)\\ & +\left(\frac{teflon\to -4.55\times 10^{-8}}{plexiglass\to 4.55\times 10^{-8}}\right)\\ & +1.07\times 10^{-7}\times \left(\frac{C_{Glycerol\;}-35}{15}\right)\\ & -7.09\times 10^{-8}\times \left(\frac{C_{MMT\;}-10.5}{9.5}\right)\\ & +\left(\frac{Rice\to -2.85\times 10^{-7}}{Chickpea\to \;2.85\times 10^{-7}}\right)\\ & +\left(\frac{C_{Starch\;}-5.5}{1.5}\right)\times \left(\frac{teflon\to -5.83\times 10^{-8}}{plexiglass\to 5.83\times 10^{-8}}\right)\\ & +\left(\frac{C_{Starch\;}-5.5}{1.5}\right)\times \left(\left(\frac{C_{MMT\;}-10.5}{9.5}\right)\times -1.06\right)\\ & +\left(\frac{C_{Starch\;}-5.5}{1.5}\right)\times \left(\frac{Rice\to -1.42\times 10^{-7}}{Chickpea\to \;1.42\times 10^{-7}}\right)\\ & +\left(\frac{teflon\to \left(\frac{C_{MMT\;}-10.5}{9.5}\right)\times -1.01\times 10^{-7}}{plexiglass\to \left(\frac{C_{MMT\;}-10.5}{9.5}\right)\times 1.01\times 10^{-7}}\right) \end{array}$$

The optimization of the film opacity index was based on the statistically significant examined functional parameters (starch, glycerol and MMT concentration, drying temperature, starch species and drying tray type). The linear regression equation (R^2^ = 0.95) is:

(5) $$\begin{array}{cc} Y=20.64+1.86 & \times \left(\frac{\left(C_{Starch}-5.5\right)}{1.5}\right)+\left(\frac{teflon\to 1.96}{plexiglass\to \;-1.96}\right)\\ & -4.22\times \left(\frac{\left(C_{glycerol}-35\right)}{15}\right)\\ & +4.54\times \left(\frac{(Drying\;Temperature-45}{15}\right)\\ & +8.84\times \left(\frac{\left(C_{MMT}-10.5\right)}{9.5}\right)+\left(\frac{Rice\to -2.7}{Chickpea\to \;2.7}\right)\\ & +\left(\frac{\left(C_{Starch}-5.5\right)}{1.5}\right)\times \left(\left(\frac{\left(C_{glycerol}-35\right)}{15}\right)\times 2.99\right)\\ & +(\left(\frac{teflon\to \left(\frac{\left(C_{glycerol}-35\right)}{15}\right)\times -4.17}{plexiglass\to \left(\frac{\left(C_{glycerol}-35\right)}{15}\right)\times 4.17}\right)\\ & +\left(\frac{\left(C_{Starch}-5.5\right)}{1.5}\right)\times \left(\left(\frac{\left(C_{Starch}-5.5\right)}{1.5}\right)\times -13.85\right)\\ & +\left(\frac{(Drying\;Temperature-45}{15}\right)\\ & \times \left(\left(\frac{(Drying\;Temperature-45}{15}\right)\times 9.85\right) \end{array}$$

**Figure 8:**
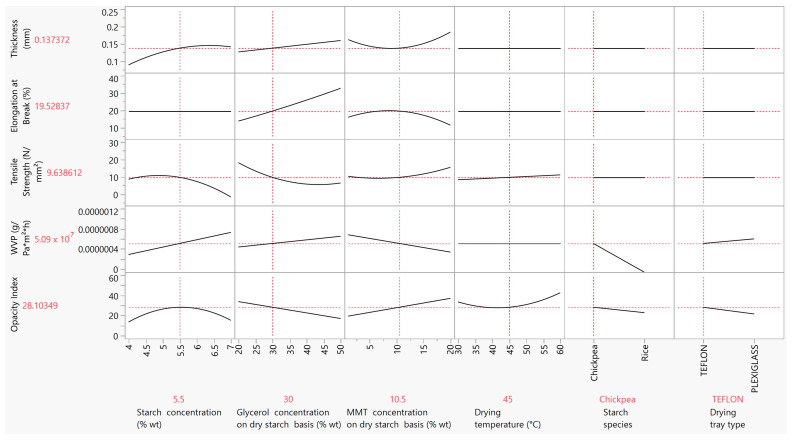
Optimization plot based on the effect of the starch, glycerol and MMT concentration, drying temperature, starch species and drying tray type on the film thickness, tensile strength, elongation at break, WVP and opacity.

The aim of the optimization and desirability analysis was to come up with the best combination of factors for obtaining desirable attributes in the final product. In starch-based biodegradable films for food packaging applications, desirability generally includes combining high tensile strength and elongation at break values, which will provide resistance and extensibility, respectively, but also low thickness values for low-cost applications, as well as high moisture barrier properties, and hence low WVP values. Therefore, the best outcome should include the maximization of the tensile strength and the elongation at break values and the minimization of thickness and WVP. The results from the optimization plot (Figure 8) in conjunction with the aforementioned aims included: 5.5% wt starch concentration, 30% wt glycerol concentration on a dry starch basis, 10.5% wt MMT concentration on a dry starch basis, 45 °C drying temperature, chickpea as the starch species and plexiglass as the drying tray type. The optimum responses with this specific combination of factors were as follows: 0.14 mm for the film thickness, 9.63 N/mm^2^ for the tensile strength, 19.53% for the elongation at break, 6 × 10^−7^ g·Pa^−1^·h^−1^·m^−1^ for the WVP and 21.41 for the opacity index. Comparing these properties to those of synthetic polymer films, the tensile strength is within the range of low-density polyethylene (LDPE) (8–20 N/mm^2^) [70], and the WVP is lower than that of LDPE (2.5 × 10 ^−8^ g·Pa^−1^·h^−1^·m^−1^) [71]. Preliminary results from ongoing research in our lab towards the preparation of films from starch inclusion complexes of antioxidant and antimicrobial substances show that both the tensile strength and the WVP can be enhanced due to the increased crystallinity of films associated with the formation of starch complexes.

### 3.6. Antimicrobial Activity

**Figure 9:**
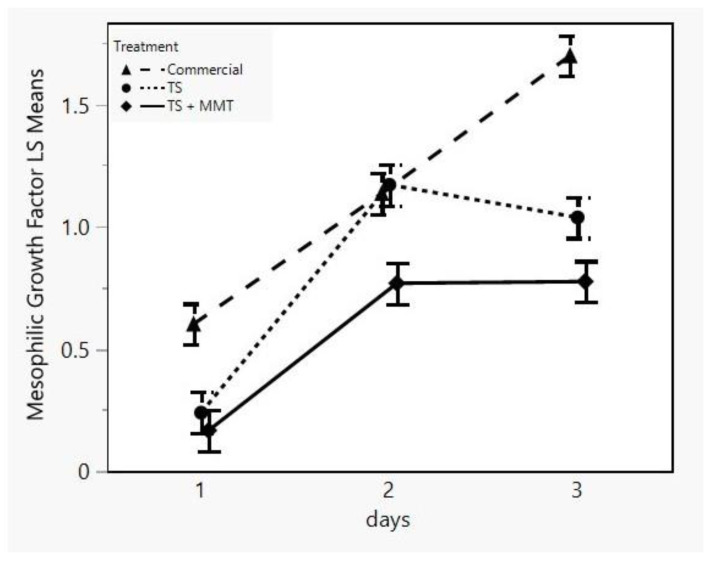
LSM plot of the mesophilic bacteria growth factor of minced meat samples in different packages (commercial = LDPE, TS = starch films, TS + MMT = starch films with MMT) versus storage time.

One of the main attributes of the concept of “active” packaging is the active contribution of the package to the protection of the product from various factors, such as environmental or spoilage and pathogen microbial factors, which cause degradation of the product and minimize its shelf life. Therefore, the incorporation of antimicrobial compounds into biodegradable starch packaging films may be an effective way of protecting the food product and prolonging its shelf life. MMT is commonly modified with quaternary ammonium salt groups, which are known to enhance antimicrobial activity [72]. Consequently, the efficacy of the biodegradable starch films containing MMT (prepared according to the optimization results) regarding their antimicrobial activity was evaluated on a perishable food (minced meat) against mesophilic and psychrotrophic bacteria during a three-day preservation period. Data showed that for mesophilic bacteria, the influence of both time and treatment and their interaction was statistically significant (*p* < 0.05) (Table 3). More specifically, the growth factor of the sample TS + MMT was lower (*p* < 0.05) at all sampling days than that of the commercial sample (Figure 9). Also, the growth factor of the sample TS + MMT was lower than that of the sample TS on days 2 and 3.

**Figure 10:**
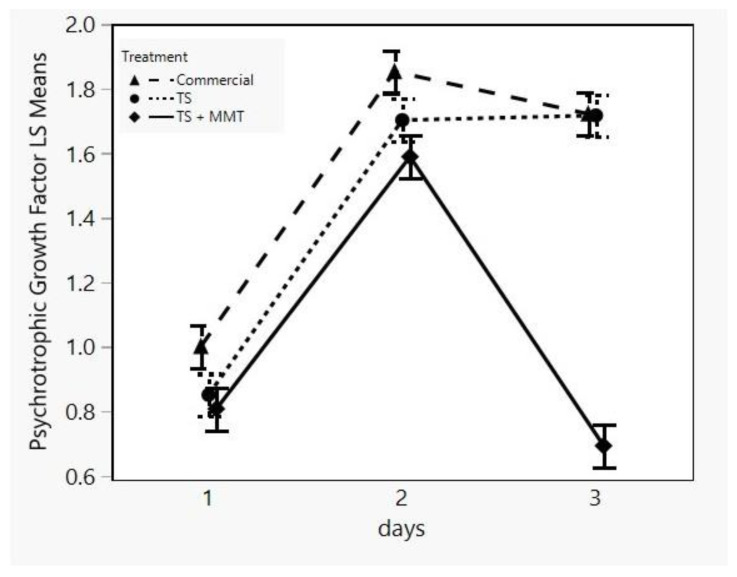
LSM plot of the psychrotrophic bacteria growth factor of minced meat samples in different packages (commercial = LDPE, TS = starch films, TS + MMT = starch films with MMT) versus storage time.

Psychrotrophic bacteria are of great interest regarding meat and meat product deterioration, because these products are mainly stored under refrigeration between 4 and 7 °C. Some common species of these bacteria are *Brochothrix thermosphacta*, *Carnobacterium* spp., Enterobacteriaceae, *Lactobacillus* spp., *Leuconostoc* spp., *Pseudomonas* spp. and *Shewanella putrefaciens.* These can cause slime production, off-odor and off-flavor production, gas production and the discoloration of meat and meat products [73]. Therefore, the main goal of the food industry regarding the quality and safety of these types of products is protection from psychrotrophic spoilage and pathogen bacteria. As can be seen in Figure 10, the psychrotrophic bacteria growth factor of the sample TS + MMT was lower during all storage days compared to both the commercial and the TS sample, particularly at day 3, where a negative trend can be observed. This implies that the psychrotrophic bacteria population of the sample TS + MMT was not only lower than its counterparts, but it also decreased. Since bacterial cell life is comprised of four stages, namely, the lag, log, stationary and death phase, a decrease in the population means cell death [74]. In the present work, this decrease may be due to the bactericidal activity of MMT as a quaternary ammonium compound (QAC), which is in accordance with the literature [75,76]. Regarding the onset of the bactericidal activity of MMT (day 3), it has been shown that the presence of Cloisite^®^ extended the lag phase of the microbial culture during biodegradation studies [77]. This observation is of high importance, despite the fact that the treatment was not significant (*p* > 0.05) according to the results of the statistical analysis (Table 4).

### 3.7. Biodegradability

**Figure 11:**
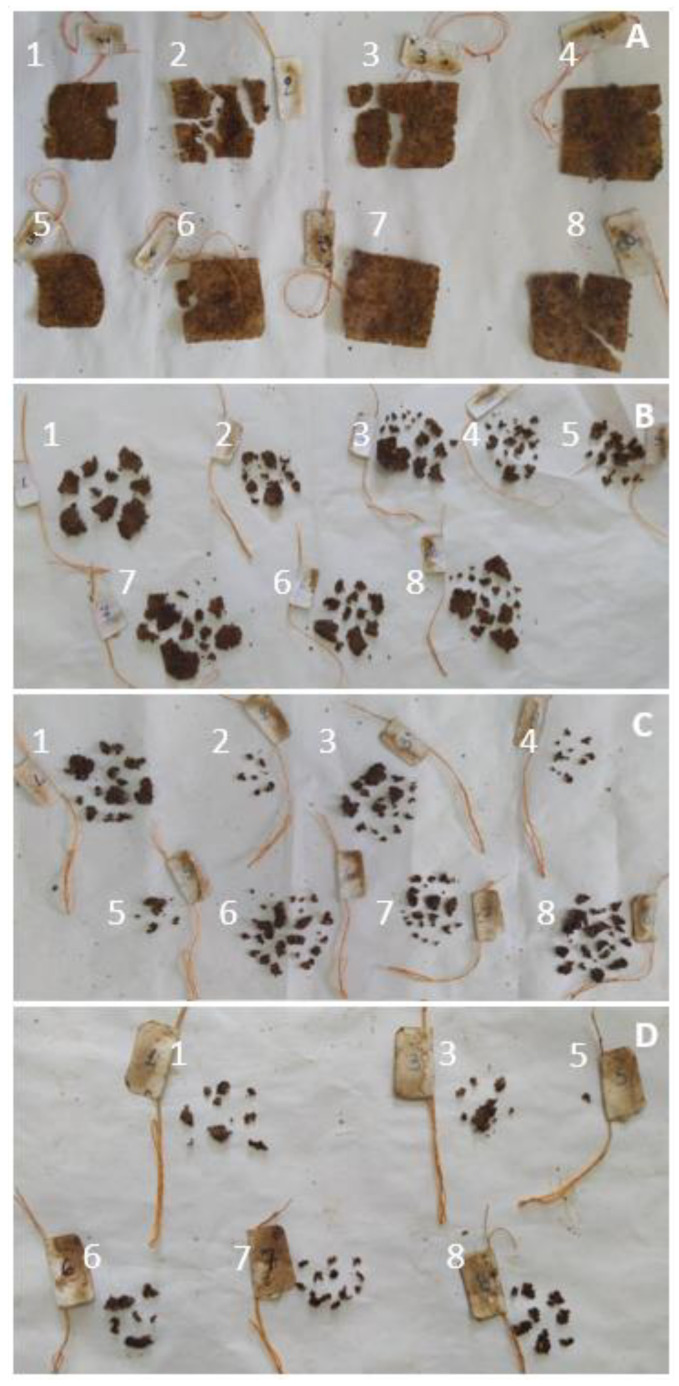
Biodegradability of starch films prepared according to the optimization results buried for thermophilic incubation under simulated composting conditions after 2 (**A**), 4 (**B**), 6 (**C**) and 8 (**D**) days. Numbers identify the films at the different timepoints.

The most important concern of synthetic polymer food packaging is the amount of produced waste and the inability of these materials to degrade. Natural materials have been proposed as an alternative due to their biodegradability. To assess this property, starch films that were prepared according to the optimization results were buried for thermophilic incubation under simulated composting conditions. Indeed, as can be seen in Figure 11, even after 2 days, some films started to break into pieces. The process was accelerated between days 2 and 4, and by day 8, it was impossible to recover some of the films. Finally, after 10 days, it was impossible to recover any of the films. It should be noted that according to the ISO 20200:2015 determination of the degree of disintegration of plastic materials under simulated composting conditions, the thermophilic incubation period lasts from a minimum of 45 to a maximum of 90 days, followed by a mesophilic incubation period with a maximum of 90 days, if, at the end of the thermophilic incubation period, the test material has not sufficiently disintegrated [41].

### 3.8. Industrial-Scale Handling

**Figure 12:**
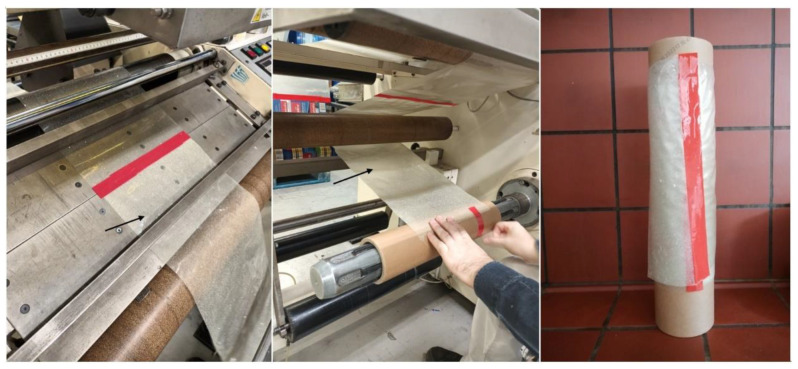
Industrial-scale handling of a biodegradable starch film prepared according to the optimization results in a rewinding machine. Black arrows indicate the biodegradable starch film.

In the literature, studies on biodegradable starch films for food packaging applications focus exclusively on the experimental development of films and the evaluation of their properties. In the present study, a large piece of starch film was prepared according to the optimization results. It should be noted that casting the film at these dimensions raised some technical issues, such as the inability to spread the viscous thermoplastic starch dispersion (which becomes more viscous as the temperature decreases) evenly on such an extended surface and the lack of appropriate equipment to produce such large quantities of thermoplastic starch dispersion. The latter demanded more vigorous agitation, which resulted in more air bubbles embedded within the film. Upon successfully overcoming these issues with the preparation of a uniform film, a piece with the appropriate dimensions was cut and tested in an industrial application, namely, handling of the film in a rewinding machine. The film was successfully rewound into a paper cylinder (Figure 12). This is a very important result that proves that these types of films can be handled by production-scale processing machines, and to the best of our knowledge, this is the first step towards their industrial application.

## 4. Conclusions

The observations from the present study represent a holistic approach to the preparation of starch biodegradable films and the quantitative and qualitative factors that affect such preparation. The optimization process (maximizing the tensile strength and elongation at break; minimizing the thickness, WVP and opacity) will be a useful tool for the industrial-scale design and production of biodegradable starch films according to their intended use. The incorporation of MMT at a level of 10.5% wt on a dry starch basis decreased the mesophilic and, most importantly, the psychrotrophic bacteria growth factor in a perishable food model, proving the antimicrobial activity and the potential of these films to be used as active food packaging materials. The biodegradability assessment showed that these films are a promising candidate for replacing synthetic polymer food packaging. Finally, the industrial-scale application of the films prepared according to the optimization results was demonstrated in terms of handling the film. In this vein, future plans include the evaluation of the lamination capability of biodegradable starch films reinforced with MMT. Since this type of packaging aims to replace conventional plastic packaging, the migration of nanoclays to food materials should be studied, because nanoclays have limited application due to the regulatory framework. However, if the nanoclay to be used is safe for human consumption, then migration should not be an issue of major concern. The cost of the raw materials for the preparation of the films is low, and starch and MMT are abundant in nature. The ability to use rice and legumes that have been rejected from the food industry as unfit for human and animal consumption as a starch source is expected to greatly contribute to the circular economy and sustainability, as well as to resolve issues related to using possible food-grade starch intended for human consumption. Although the results presented in this study are promising for the development of a fully biodegradable packaging material, further research is needed before industrial implantation, particularly regarding the manufacturing line of film production. Casting will not be applicable in this scenario, and other alternatives, such as extrusion and calendering, should be studied. This is a subject of ongoing research in our laboratory.

## Figures and Tables

**Table 1:** Experimental values and coded levels of the independent variables used for the central composite design.

Variable	Coded Variable Levels (DSD)		
	−1	0	1
Starch concentration (% wt)	4	5.5	7
Glycerol concentration on dry starch basis (% wt)	20	35	50
MMT concentration on dry starch basis (% wt)	1	10.5	20
Drying temperature (°C)	30	45	60
Starch species	Chickpea	Rice	
Drying tray type	Plexiglass	Teflon	

**Table 2:** Experimental values of the independent variables used for the central composite design for each test run.

Test Number	Starch Concentration (% wt)	Glycerol Concentration On Dry Starch Basis (% wt)	MMT Concentration on Dry Starch Basis (% wt)	Drying Temperature (°C)	Starch Species	Drying Tray Type
1	4	20	10.5	60	chickpea	plexiglass
2	5.5	35	10.5	45	chickpea	teflon
3	7	20	20	30	chickpea	teflon
4	4	20	20	30	rice	plexiglass
5	5.5	35	10.5	45	rice	plexiglass
6	7	50	1	60	chickpea	teflon
7	7	50	10.5	30	rice	teflon
8	4	50	1	60	rice	plexiglass
9	7	35	20	60	rice	plexiglass
10	4	35	1	30	chickpea	teflon
11	7	20	1	60	rice	teflon
12	4	50	20	60	rice	teflon
13	7	20	1	30	chickpea	plexiglass
14	5.5	50	1	30	rice	plexiglass
15	4	50	20	30	chickpea	plexiglass
16	7	50	20	45	chickpea	plexiglass
17	5.5	20	20	60	chickpea	teflon
18	4	20	1	45	rice	teflon

**Table 3:** Statistical analysis regarding mesophilic bacteria (* denotes statistical significance).

Source	SS	MS Num	DF Num	F Ratio	Prob > F
time	2.3848	1.1924	2	50.3951	<0.0001 *
treatment × time	0.32236	0.08059	4	34.2253	0.0003 *
treatment	0.21683	0.10841	2	49.2343	<0.0001 *
subjects (treatment) and random	0.00569	0.0019	3	0.8055	0.5351

**Table 4:** Statistical analysis regarding psychrotrophic bacteria (* denotes statistical significance).

Source	SS	MS Num	DF Num	F Ratio	Prob > F
time	2.08921	1.0446	2	717.3254	<0.0001 *
treatment × time	0.6966	0.17415	4	119.5880	<0.0001 *
treatment	0.04136	0.02068	2	2.6390	0.1893
subjects (treatment) and random	0.06179	0.0206	3	14.1430	0.0040 *

## Data Availability

The data used to support the findings of this study can be made available by the corresponding author upon request.
